# Reassignment of *Drosophila willistoni* Genome Scaffolds to Chromosome II Arms

**DOI:** 10.1534/g3.115.021311

**Published:** 2015-10-04

**Authors:** Carolina Garcia, Alejandra Delprat, Alfredo Ruiz, Vera L. S. Valente

**Affiliations:** * Departamento de Genética, Instituto de Biociências, Universidade Federal do Rio Grande do Sul, Brazil 15053; † Departament de Genètica i de Microbiologia, Facultat de Biociències, Universitat Autònoma de Barcelona, Barcelona, Spain 08193

**Keywords:** *Drosophila willistoni*, polytene chromosome II, physical markers, *in situ* hybridization, Muller elements

## Abstract

*Drosophila willistoni* is a geographically widespread Neotropical species. The genome of strain Gd-H4-1 from Guadeloupe Island (Caribbean) was sequenced in 2007 as part of the 12 *Drosophila* Genomes Project. The assembled scaffolds were joined based on conserved linkage and assigned to polytene chromosomes based on a handful of genetic and physical markers. This paucity of markers was particularly striking in the metacentric chromosome II, comprised two similarly sized arms, IIL and IIR, traditionally considered homologous to Muller elements C and B, respectively. In this paper we present the cytological mapping of 22 new gene markers to increase the number of markers mapped by *in situ* hybridization and to test the assignment of scaffolds to the polytene chromosome II arms. For this purpose, we generated, by polymerase chain reaction amplification, one or two gene probes from each scaffold assigned to the chromosome II arms and mapped these probes to the Gd-H4-1 strain’s polytene chromosomes by nonfluorescent *in situ* hybridization. Our findings show that chromosome arms IIL and IIR correspond to Muller elements B and C, respectively, directly contrasting the current homology assignments in *D. willistoni* and constituting a major reassignment of the scaffolds to chromosome II arms.

*Drosophila willistoni* belongs to the *willistoni* species group of the subgenus *Sophophora*. With a widespread geographic distribution in the Neotropical region (Val *et al.* 1981; Bächli 2015), it is one of the most polymorphic species for chromosomal inversions in the entire genus *Drosophila* (Sperlich and Pfriem 1986; Rohde and Valente 2012) and is a classic model organism for research in population and evolutionary genetics, speciation, ecological genetics, and molecular evolution (Ehrman and Powell 1982; Krimbas and Powell 1992; Markow and O’Grady 2007). The karyotype of this species consists of two metacentric chromosomes (X and II) and a rod chromosome (III) (Dobzhansky and Pavan 1943).

Muller (1940) proposed that, in *Drosophila*, the genic content of chromosome arms has been conserved despite numerous rearrangements that have occurred during the evolution of the genus, a notion that has been supported widely in subsequent studies (Bhutkar *et al.* 2008) . The current chromosomal arm homologies for *D. willistoni* are as follows (Schaeffer *et al.* 2008): XL and XR are homologous to *D. melanogaster* X and 3L (Muller elements A and D), IIL and IIR are homologous to *D. melanogaster* 2R and 2L (Muller elements C and B), whereas chromosome III is homologous to *D. melanogaster* 3R and 4 (Muller elements E and F).

The genome of *D. willistoni* (strain Gd-H4-1, Guadeloupe Island, Caribbean) was completely sequenced and assembled in 2007 by the *Drosophila* 12 Genomes Consortium. The scaffolds were joined based on conserved linkage in the orthologous genes located at the scaffold ends (Schaeffer *et al.* 2008). Subsequently, scaffolds were anchored to the polytene chromosomes based on a handful of genetic markers, mainly enzyme loci and other genes taken from classical studies (Ayala *et al.* 1972; Lakovaara and Saura 1972; Dobzhansky and Powell 1975), and less than 10 gene markers mapped by *in situ* hybridization (ISH). There is scarcity of gene markers occurring in the metacentric chromosome II, which shows a high level of segregating inversions, mainly in the IIL arm (Rohde and Valente 2012). This chromosome has two similarly sized arms named IIL and IIR (Dobzhansky 1950), which have been traditionally considered homologous to Muller elements C and B, respectively (Lakovaara and Saura 1972; review in Sorsa 1988). Eight scaffolds were assigned to each of the two arms of this chromosome in the Gd-H4-1 strain, based on linkage information for five genetic markers and the physical mapping of another two markers. Thus, the current assignation of scaffolds to *D. willistoni* chromosome II arms should be viewed with caution and as provisional, as stated by Schaeffer *et al.* (2008).

To increase the number of markers mapped by ISH and to test the assignment of scaffolds to chromosome II in strain Gd-H4-1, we mapped 18 new markers for chromosome II by using nonfluorescent ISH. Additionally, three gene markers were physically mapped to chromosome X and one gene marker mapped to chromosome III, constituting a total of 22 new gene markers.

## Materials and Methods

### *Drosophila* stock and cytological preparations

The strain of *D. willistoni* used in this study was Gd-H4-1. This strain comes from Guadeloupe Island in the Caribbean, and it was used for sequencing the complete genome of this species (*Drosophila* 12 Genomes Consortium 2007). For that purpose, the high degree of polymorphism for inversions naturally present in this species (Rohde and Valente 2012) was virtually solved by successive inbreeding (*Drosophila* 12 Genomes Consortium 2007). The stock was purchased from the UC San Diego Stock Center and maintained at 21° in standard cornmeal−agar−yeast culture medium.

The cytological preparations of polytene chromosomes for the nonfluorescent ISH were made with salivary glands from larvae in the third stage of development fed with extra fresh yeast. Salivary glands were dissected in saline solution and immediately transferred to a coverslip with a drop of 45% acetic acid for 3 min for fixation of the glands. Then, the excess of acetic acid was removed, and a drop of acetic acid:water:lactic acid mixture in 3:2:1 (v/v) ratio was added and rested for 4 min. The material was then placed on a slide, spread, and squashed. All the analyses of chromosomal preparations were done on a phase contrast NIKON Optiphot-2- microscope.

### Gene probes

Release 1.04 of genome of *D. willistoni* available in FlyBase database (St. Pierre *et al.* 2014), was used to generate 22 gene probes. Two probes were designed on scaffolds 4577 (IIL) and 4558 (IIR); for the rest of the chromosome II scaffolds, only one gene was chosen (Table 1). Additionally, three probes were designed for chromosome X and one for chromosome III (supporting information, Table S1). All the polymerase chain reaction (PCR) primers were designed with Primer Designer v.1.01 (Scientific and Educational Software).

**Table 1:** Gene markers used for scaffold reassignment to *Drosophila willistoni* chromosome II listed from centromere to telomere

*D. willistoni* Gene	*D. melanogaster* Ortholog Gene	Scaffold Number	Scaffold Orientation	Scaffold Position of Gene	Cytological Position	Primers F and R (5′-3′)
IIL arm (Muller B element)						
*Dwil\GK18743*	*Dmel\Lamp1*	4884	+	878,652.0.883,516	37B	GTCAAGCAGTAGCAGCACCA
						GCCACGCGAAGTTGATCGAC
*Dwil\GK24519*	*Dmel\CG9515*	4585	+	282,915.0.284,232	38A	ATTCAATTCACAGCACAACC
						GGACTCAATGCGGAACTATG
*Dwil\GK18260*	*Dmel\Yuri*	4577	−	1,906,481.0.1,918,823	45A	GTGAAGAGCCTACACACAGC
						CTTCTGAGATGATCCACGAC
*Dwil\Adh*	*Dmel\Adh*	4577	−	2,980,068.0.2,982,042	44A	CATGGAACGTGTTAAGTGCC
						AGTTCACAGCAATGGTACGC
*Dwil\GK23840*	*Dmel\CG17549*	4516	+	60,623.0.62,773	46A	GGAGGATATGCTGGTGGTTA
						GTGCTGACTTGCTCCAACTG
*Dwil\GK15054*	*Dmel\CG7371*	4521	+	716,961.0.719,512	47A	ATCCTGAGCCTGAGTTCCAC
						CGCCAAGAGAATTGTCATCG
*Dwil\GK12721*	*Dmel\CG13127*	4752	+	52,463.0.54,862	55A	GCAGCTCGATGAACTCTATG
						TCTCCGAAGACTGTGTACTC
*Dwil\GK18432*	*Dmel\fusl*	4945	+	417,864.0.419,442	55A	GCATCAGCCTCATATCCATC
						GTCAACACTCTCGGCTCCAG
*Dwil\GK21099*	*Dmel\Rab3-GAP*	4851	−	226,612.0.230,981	55C	CTGGAGCAGTCAAGGCGAGA
						ATCCAAGCATCCTAAGCGTG
IIR arm (Muller C element)						
*Dwil\GK23049*	*Dmel\bw*	4954	+	2,790,903.0.2,793,354	56C	CAGTAGTAACCACTCCGATG
						GCGGACACATTGTCTACCAG
*Dwil\GK22144*	*-*	4558	+	73,772.0.80,277	58C	CATTCGACGATCTCAGCAAC
						TCACTTCGGACTACTCCAGC
*Dwil\GK22138*	*Dmel\px*	4558	+	211,240.0.284,571	59B	GCTGCATTAGATCCTCATAG
						GGCAGCCAACAGTCCATACA
*Dwil\vlc*	*Dmel\vlc*	4382	−	1,240,131.0.1,244,696	62A	CAACGCCACTATCTGTGAAG
						TCTCATTGCACTCACCTACA
*Dwil\GK17912*	*Dmel\FLASH*	4822	+	150,635.0.154,598	64B	ACGATGATCTGGATGAGTTC
						TACAACATACCTAGTTCCGC
*Dwil\GK20645*	*Dmel\CG2269*	4510	−	3,566,941.0.3,573,564	68C	CTGATGGACACCACAGAGTG
						ACTCGTACAACATGGCGGAC
*Dwil\GK15808*	*Dmel\ Ir60a*	4514	−	205,020.0.207,611	71A	AACGAGGCAGTCACCGATAC
						CATATCGGACGCTCTTGAAC
*Dwil\Adam*	*Dmel\Adam*	4513	+	22,848.0.24,439	72A	GGTGAGGATGACGATGAGGA
						TCCGAATGTAAGAGCTCCAC
*Dwil\GK19495*	*Dmel\Hsf*	4512	+	1,798,077.0.1,801,918	77A	GGCTACCGTCATAAGATCAG
						AGAACATACGTGGACGTCAG

The scaffold number corresponds to the last four numbers of the scaffolds, which all start with scf2_110000000. The scaffold number and scaffold position of genes correspond to the material available in the FlyBase database (St. Pierre *et al.* 2014). The scaffold orientation is according to Schaeffer *et al.* (2008)

PCR amplifications were performed with Taq DNA Polymerase, recombinant (Invitrogen) following the manufacturer’s instructions. PCR products were purified with Exonuclease I (USB) and Shrimp Alkaline Phosphatase (USB) and sequenced by Macrogen INC (Seoul, Korea). All sequences confirmed by BLASTN in the FlyBase database (St. Pierre *et al.* 2014).

Probes were labeled with a Biotin PCR Labeling Core Kit (Jena Bioscience) per the manufacturer instructions, with minor adjustments. The labeled products were purified with a NucleoSpin Gel and PCR Clean-up Kit (MACHEREY-NAGEL).

### ISH and mapping

Nonfluorescent ISHs were carried out at 37° using 100−150 ng of biotin-labeled probe on each chromosomal slide, following the protocol of Biémont *et al.* (2004) with minor modifications: 1) The slides were washed in 70% ethanol before the later washing with 95% ethanol in the pre-hybridization step; 2) two washes with 2× saline sodium citrate at 37° for 10 min and one wash for a few seconds in 2× saline sodium citrate at room temperature after the hybridization step; 3) one wash with 1× phosphate-buffered saline (PBS) just after the washes with 0.1% Triton in 1× PBS after the detection step; 4) one wash with 0.1% Triton in 1× PBS before the wash with 1× PBS after the revelation step.

Hybridization signals were detected and revealed using a Vectastain ABC KIT (Vector Laboratories) and DAB Substrate (Roche), respectively. Then, the slides were stained with Giemsa solution (5%) for 5 min and made permanent with EUKITT (Panreac). Finally, the positives hybridization signals were mapped in the polytene chromosome of the sequenced strain Gd-H4-1 and recorded with a Moticam Package (Moticam3 with 3.0 MP and Motic Images Plus 2.0). Their physical locations were determined according to current photomap of the species (Schaeffer *et al.* 2008) and the chromosomal analysis of this strain by Rohde and Valente (2012**)**.

### Data availability

All cytological materials are available for viewing at *Drosophila* Laboratory of Universidade Federal do Rio Grande do Sul, Brazil. More detailed data are available upon request.

## Results and Discussion

**Figure 1:**
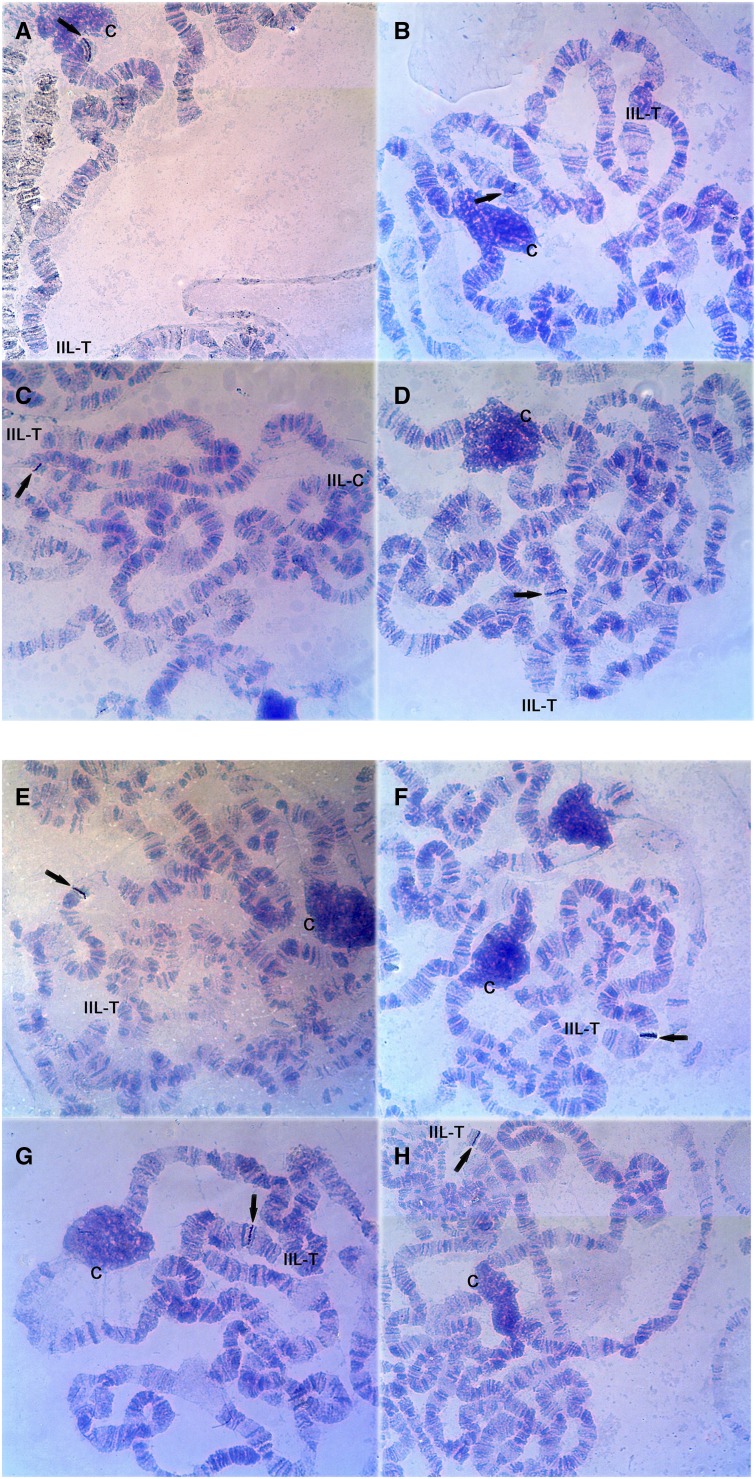
Gene probes hybridized to the *D. willistoni* chromosome IIL arm ordered from centromere to telomere (A) *Dwil\GK18743* (scaffold 4884); (B) *Dwil\GK24519* (scaffold 4585); (C) *Dwil\GK18260* (scaffold 4577); (D) *Dwil\GK23840* (scaffold 4516); (E) *Dwil\GK15054* (scaffold 4521); (F) *Dwil\GK12721* (scaffold 4752); (G) *Dwil\GK18432* (scaffold 4945); and (H) *Dwil\GK21099* (scaffold 4851). The black arrows indicate hybridization signals. IIL-T, IIL arm telomere; IIL-C, IIL arm centromere; C, chromocenter.

**Figure 2:**
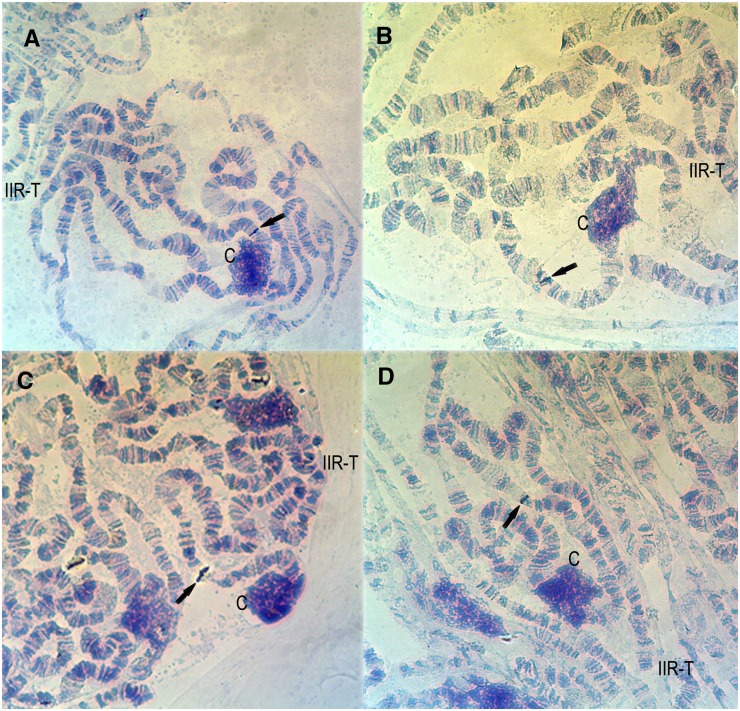

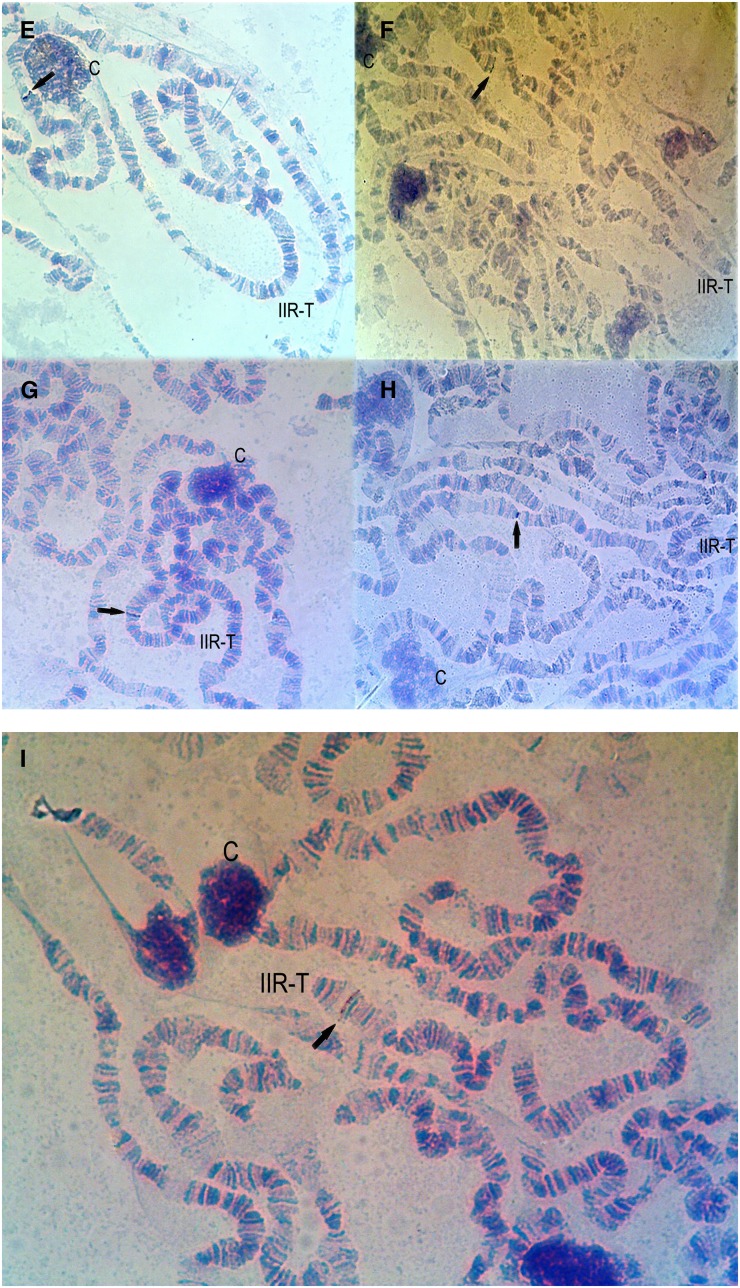
Gene probes hybridized to *D. willistoni* IIR arm ordered from centromere to telomere (A) *Dwil\GK23049* (scaffold 4954); (B) *Dwil\GK22144* (scaffold 4558); (C) *Dwil\GK22138* (scaffold 4558); (D) *Dwil\vlc* (scaffold 4382); (E) *Dwil\GK17912* (scaffold 4822); (F) *Dwil\GK20645* (scaffold 4510); (G) *Dwil\GK15808* (scaffold 4514); (H) *Dwil\Adam* (scaffold 4513); and (I) *Dwil\GK19495* (scaffold 4512). The black arrows indicate hybridization signals. IIR-T, IIR arm telomere; C, chromocenter.

With a stringency of approximately 77% (Schwarzacher and Heslop-Harrison 2000) and considering the use of homologous probes, each gene probe mapped in this study showed a single hybridization signal in the polytene chromosomes of *D. willistoni*. Remarkably, all probes designed for scaffolds previously assigned to the IIL chromosome arm hybridized to the IIR chromosome arm. In turn, all probes designed for scaffolds previously assigned to the IIR chromosome arm hybridized to the IIL chromosome arm. In both arms, the scaffolds are currently ordered from centromere (scaffold 1) to telomere (scaffold 8) (Schaeffer *et al.* 2008). In the IIL arm, our results indicate that the same order of scaffolds from centromere to telomere holds (Table 1 and Figure 1). In contrast, in the IIR arm, our results show that the order is reversed, with scaffold 8 located near the centromere and scaffold 1 located near the telomere (Table 1 and Figure 2). Our analysis corroborated these orientations in the two cases where two gene probes were mapped for the same scaffold (scaffold 4577 in the IIL arm and scaffold 4558 in the IIR arm; see Table 1).

In the case of the IIR arm, Schaeffer *et al.* (2008) inferred that scaffold 4558 has a negative orientation. According to their study, the cytological position of the *GK22138* gene would appear before that of the other hybridized gene, *GK22144*. As seen in Figure 2, B and C and in Table 1, our hybridizations resulted in the GK22144 gene being positioned before the GK22138 gene. A comparison of our results with the previous observations used for anchoring scaffolds (Schaeffer *et al.* 2008) seems appropriate. One caveat should be pointed out from the beginning: the variability of gene arrangements within *D. willistoni* makes linkage mapping an arduous task. Mutants have been often isolated or generated from different strains and crossings to map those mutants may involve inversions hindering or reducing recombination. Additionally, chromosomal differences between populations may lead to different results when using different strains. Spassky and Dozhansky (1950) mapped six mutants to chromosome II of *D. willistoni* (*bw*, *abb*, *px*, *hk*, *pw*, *Sv*, and *S*). Most of these mutants were obtained from a strain from Belem (Pará, Brazil), but some (*e.g.*, *bw* and *hk*) were generated in X-rayed cultures from strains of a different origin. The eye-color mutant *brown* (*bw*) was located at one end of the chromosome and was considered the starting point of the genetic map. However, Spassky and Dozhansky (1950) gave no indication as to the correspondence between chromosome II arms and Muller elements. An addition to this map was made later by Lakovaara and Saura (1972), who determined the linkage map location of four chromosome II enzyme loci (*Est-5*, *Gpdh*, *Mdh-2*, and *Adh*). Most of the *D. willistoni* stocks used by them originated in the Leeward Islands (West Indies). They were the first to report that *Est-5*, *Gpdh*, *Mdh-2*, and *Adh* are located in the right arm of *D. willistoni* chromosome II and that this arm corresponds to *D. melanogaster* arm 2L (Muller element B).

To anchor the scaffolds to *D. willistoni* chromosome II, Schaeffer *et al.* (2008) used linkage information from five genetic markers (*bw*, *px*, *hk*, *Gpdh*, and *Adh*) and ISH information from two other markers (*Adh* and *Cl*). The five genetic markers were chosen to maximize reliability but were taken from two different studies. The locations of the five markers in the combined linkage map are as follows: *bw* (0 cM) – *px* (28 cM) – *hk* (31 cM) – *Gpdh* (59 cM) – *Adh* (66 cM). The first two markers were assigned to chromosome arm IIL, whereas the latter three were assigned to chromosome arm IIR. However, in addition to the fact that the strains used by Lakovaara and Saura (1972) were different from those used by Spassky and Dobzhansky (1950), they were different from the *D. willistoni* sequenced strain from Guadeloupe Island.

We have hybridized PCR probes for the three genes considered by Schaeffer *et al.* (2008): *brown* (*bw*), *plexus* (*px*), and *Alcohol dehydrogenase* (*Adh)*. In *D. willistoni*, the orthologous genes for *D. melanogaster bw* and *px* are named *Dwil\GK23049* and *Dwil\GK22138*, respectively, in the FlyBase database (St. Pierre *et al.* 2014). Our ISH analysis mapped *Dwil\GK23049* and *Dwil\GK22138* to sections 56C and 59B of the *D. willistoni* chromosome IIR arm (Table 1, Figure 2, A and C). Thus, *bw* and *px* are located in the sequenced *D. willistoni* strain in arm IIR near the centromere and not in the tip of arm IIL as assumed by Schaeffer *et al.* (2008). Because these two genes also served as a basis for the orientations of the scaffolds, these findings reinforce the idea that scaffolds of IIR arm are in the opposite orientation to the one first proposed by Schaeffer *et al.* (2008).

**Figure 3:**
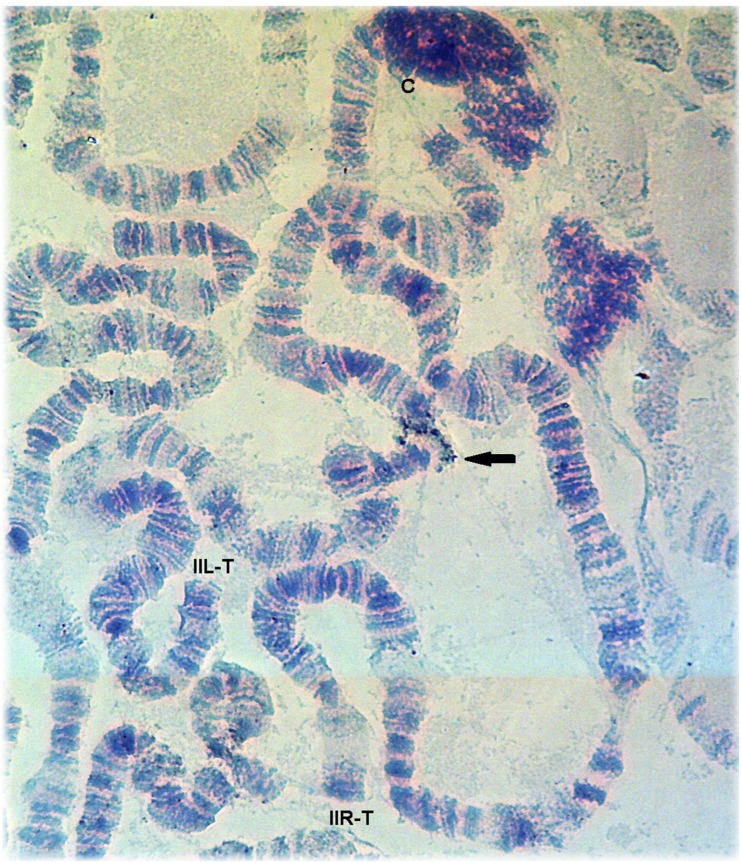
*In situ* hybridization of the gene probe of *Adh* (scaffold 4577) in *Drosophila willistoni*. In the present study, this gene hybridized to section 44A in chromosome arm IIL, in contrast to what was reported in previous studies (Lakovaara and Saura 1972; Rohde *et al.* 1995). The image shows chromosome II with the two arms IIL and IIR intact for analysis. The black arrow indicates the site of the single hybridization signal. IIL-T, IIL arm telomere; IIR-T, IIR arm telomere; C, chromocenter.

Furthermore, we mapped the *Adh* gene unequivocally to section 44A in arm IIL (Figure 3), far away from the positions in arm IIR suggested by both the linkage map and the physical localization. Because this was one of the few genes physically localized before *D. willistoni* genome sequencing, it played a key role in the anchoring of scaffolds to *D. willistoni* chromosomes and thus deserves further comment. As stated previously, *Adh* was first assigned to IIR arm by Lakovaara and Saura (1972) by linkage mapping. Rohde *et al.* (1995) in turn mapped the *Adh* gene using ISH to the IIR arm in seven species of the *willistoni* species group, including *D. willistoni*. This analysis was done with a cloned segment of DNA from *D*. *melanogaster*, probe SAC-PAT (Moses 1986). Because this probe presented multiple hybridization signals on the chromosomes of *D. willistoni*, the inference of the main physical location was made by quantification. Hence, of 28 chromosome IIR arms analyzed in that study for this species, ∼18% indicated hybridization of the *Adh* gene probe consistently in section 67 of this chromosomal arm. Given the previous observation by Lakovaara and Saura (1972), these findings were interpreted as further evidence that the IIL and IIR arms of *D. willistoni* would be equivalent to the 2R and 2L arms of *D. melanogaster*, respectively (V. L. S. Valente, personal communication). Given the importance of this gene for scaffold assignment in *D. willistoni*, we must emphasize that our technique with a specific gene probe, high stringency, and the presence of a single positive hybridization signal in the chromosomes resulted in a reliable determination of its physical location (Figure 3).

**Figure 4:**
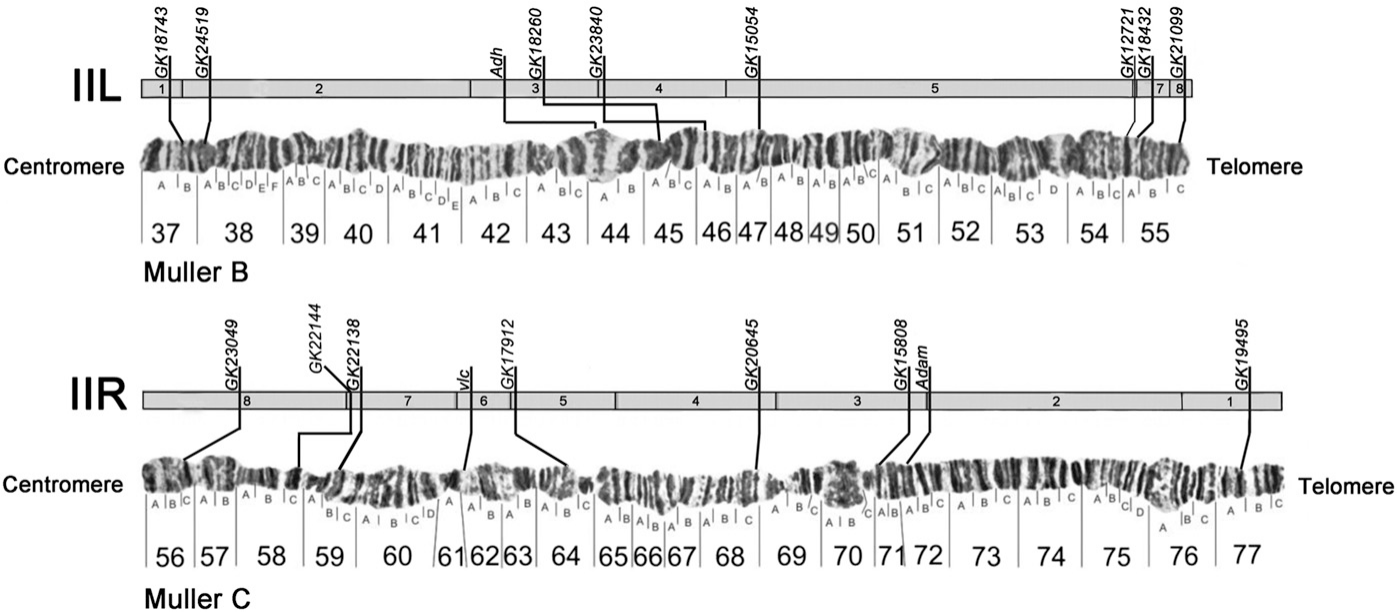
Reassignments of the sequence scaffolds and gene markers in chromosome II of *Drosophila willistoni* (Schaeffer *et al.* 2008) according to this study (Table 1). Numbers 1−8 in chromosome arm IIL correspond to scaffolds 4884, 4585, 4577, 4516, 4521, 4752, 4945, and 4851 in Table 1. Numbers 1−8 in chromosome arm IIR correspond to scaffolds 4512, 4513, 4514, 4510, 4822, 4382, 4558, and 4954 in Table 1. The position of the genes on the scaffolds matches closest position possible in the scale of genome.

Our results change the assignment of *D. willistoni* chromosome II arms to Muller elements, such that the IIL chromosomal arm is homologous to *D. melanogaster* 2L (Muller element B) and the IIR chromosomal arm is homologous to *D. melanogaster* 2R (Muller element C) (Figure 4). This result is contrary to what is traditionally accepted. The paucity of genetic markers and the ambiguous interpretation from the classic studies with crossings of those highly polymorphic species have certainly been the determinants for maintaining this mistake for so long.

In addition to aforementioned, the gene markers of *D. willistoni* were established in different populations of its wide overall geographical distribution. Because of the high degree of paracentric inversions present in this species, the order of the genetic and physical markers already established in many cases differs from that occurring in the Gd-H4-1 lineage (Schaeffer *et al.* 2008), which emphasizes the importance of establishing physical markers in the sequenced lineage and correctly aligning their genomic and cytological maps.

In view of this, to briefly examine the assignment of scaffolds to the other chromosomes, we performed additional ISHs of three genes of the X chromosome (one probe in the XL arm and two in the XR arm) and one gene on chromosome III of *D. willistoni*.

In chromosomal arm XL, a probe was designed for the gene *Dwil\GK16707*. This gene is orthologous to *Dmel\unc* in *D. melanogaster* and was chosen because it is located in the most telomeric scaffold (4963) of this arm in *D. willistoni*. In this study, its physical position was in section 1C (Figure S1), the most centromeric section in the XL arm. This indicates a disagreement with the position of this gene established in the initial assembly of the scaffold. Note that the *Dmel\unc* gene in *D. melanogaster* maps in the region 19F1 of the X chromosome, also next to the centromere.

As reported by Schaeffer *et al.* (2008), scaffold 4822 of the IIR arm (Table 1 and Table S1) had an assembly error, and a small part of it exhibited homology with Muller element D and thus belonging to chromosomal arm XR. We designed a probe for gene *Dwil*\*GK17758* that is located in this small part of scaffold 4822; that probe hybridized in section 27C of arm XR as expected (Table S1 and Figure S2). The other gene physically mapped in this arm was *Dwil\GK16749*. This gene is located in the most telomeric scaffold of this arm (4511) and was mapped to section 34B (Figure S3). Thus, our results for the chromosome XR arm are fully congruent with the scaffold assignments that Schaeffer *et al.* (2008) established on the basis of linkage information for six genetic markers and ISH information for another five genes (*E74*, *E75*, *Sod*, *Hsp83*, and *Hsp27*), a relatively high number of markers in comparison to chromosome arm XL and chromosome II.

A dot chromosome is absent in *D. willistoni*, and chromosome III was hypothesized by Sturtevant and Novitski (1941) to have originated from the fusion of Muller elements E and F. Years later, Papaceit and Juan (1998) mapped three genes in *D. willistoni* by ISH, *cubitus interrupitus* (*ci*), *ankyrin* (*Ank*), and *eyeless* (*ey*), which are located in the *D. melanogaster* dot chromosome (Muller element F). They located the three genes in the centromeric region of *D. willistoni* chromosome III, corroborating with molecular techniques, the fusion of Muller elements E and F. This event has already been confirmed for all *willistoni* subgroup species (Powell *et al.* 2011; Pita *et al.* 2014) with a timing of occurrence estimated at 15 Mya. In view of the good determination of the physical markers for the base of this chromosome we carried out an ISH with the gene *Dwil\GK22422* located in scaffold 4921 (Table S1), the most telomeric region. This gene was mapped to section 99D (Figure S4) confirming the localization of this gene in this scaffold.

In general, the physical markers used in this study for chromosomes X and III of *D. willistoni* suggest an assignment of scaffolds in these chromosomes without major relocations of genomic content compared with those observed in the case of the Muller elements B and C. However, the sequenced Gd-H4-1 strain still needs better resolution for these chromosomes by establishing physical markers from ISH as presented here for chromosome II.

This study contributes to a more accurate characterization of the genome of *D. willistoni*, providing a better basis for further genetic studies associated with it. As *D. willistoni* is an intriguing model for chromosomal and evolutionary studies, in particular, the results described here will help in analyses of interspecific chromosome evolution and genome evolution (as in Ruiz *et al.* 1997; Ranz *et al.* 2001; Papaceit *et al.* 2006 with other species) and also in the characterization of chromosomal inversion breakpoints (as in Delprat *et al.* 2009).

## Supplementary Material

Supporting Information
