# Supplementary material for: Reassignment of Drosophila willistoni Genome Scaffolds to Chromosome II Arms
Source: G3 (Bethesda). 2015 Oct 4;5(12):2559–66. doi: 10.1534/g3.115.021311 (PMC4683629; doi:10.1534/g3.115.021311)
Supplement: Supporting Information [file supp_5_12_2559__index.html]

Reassignment of Drosophila willistoni Genome Scaffolds to Chromosome II Arms — Supporting Information 

# Reassignment of *Drosophila willistoni* Genome Scaffolds to Chromosome II Arms

## Supporting Information for Garcia et al., 2015

**Files in this Data Supplement:**

- Supporting Information - Figure S1-S4 and Table S1 (PDF, 1 MB)
- Figure S1 - *In situ* hybridization of the *Dwil\GK16707* gene (scaffold 4963) to the *D. willistoni* chromosome XL arm (PDF, 415 KB)
- Figure S2 - *In situ* hybridization of the *Dwil\GK17758* gene to the *D. willistoni* chromosome XR arm (PDF, 464 KB)
- Figure S3 - *In situ* hybridization of the *Dwil\GK16749* gene (scaffold 4511) (PDF, 459 KB)
- Figure S4 - *In situ* hybridization of the *Dwil\GK22422* gene (scaffold 4921) to chromosome III (PDF, 426 KB)
- Table S1 - Gene markers used for chromosomes X and III of *Drosophila willistoni* (PDF, 360 KB)
