# Supplementary material for: Reassignment of Drosophila willistoni Genome Scaffolds to Chromosome II Arms
Source: G3 (Bethesda). 2015 Oct 4;5(12):2559–66. doi: 10.1534/g3.115.021311 (PMC4683629; doi:10.1534/g3.115.021311)
Supplement: Supporting Information [file supp_g3.115.021311_021311SI.pdf]

REASSIGNMENT OF *DROSOPHILA WILLISTONI* GENOME SCAFFOLDS TO CHROMOSOME II ARMS

Carolina Garcia[*§], Alejandra Delprat[§], Alfredo Ruiz[§], Vera L S Valente[*]

* Departamento de Genética, Instituto de Biociências, Universidade Federal do Rio Grande do Sul, Brazil 15053

§ Departament de Genètica i de Microbiologia, Facultat de Biociències, Universitat Autònoma de Barcelona, Barcelona, Spain 08193

Corresponding autor: Vera L S Valente, Departamento de Genética, Av. Bento Gonçalves, 9500, 43323M/210, Postal Code: 91501-970 - Porto Alegre, RS, Brasil, Phone (55-51) 3308-6713. E-mail: 00004887@ufrgs.br

Table S1 Gene markers used for chromosomes X and III of *Drosophila willistoni*. The scaffold number corresponds to the last four numbers of the scaffolds, which all start with scf2_110000000. The scaffold number and scaffold position of genes corresponds to the material available in the FlyBase database (St. Pierre *et al*. 2014).

***D. willistoni* Gene**	***D. melanogaster* Ortholog Gene**	**Scaffold number**	**Scaffold position of gene**	**Cytological position/chromosome**	**Primers F and R (5'-3')**
Dwil\GK16707	*Dmel\unc*	4963	432,088..435,746	1C/XL arm	ACTCAGTCTTCGACGGAAGC<br>AGTTGTATCGGATTCTACCA
Dwil\GK17758	*Dmel\ida*	4822	3,033,141..3,041,719	27C/XR arm	GCTGCATTAGATCCTCATAG<br>GGCAGCCAACAGTCCATACA
Dwil\GK16749	*Dmel\CG13313*	4511	7,841,949..7,843,999	34B/XR arm	GCTATCAGTCACCGTGTAGA<br>GGCAGTTGCTCCACCATCAC
Dwil\GK22422	*Dmel\CG31204*	4921	3,260,674..3,262,239	99D/chromosome III	GAGTCAATGCGTCCATACCA<br>GGATAATCCTCACGAGACTG

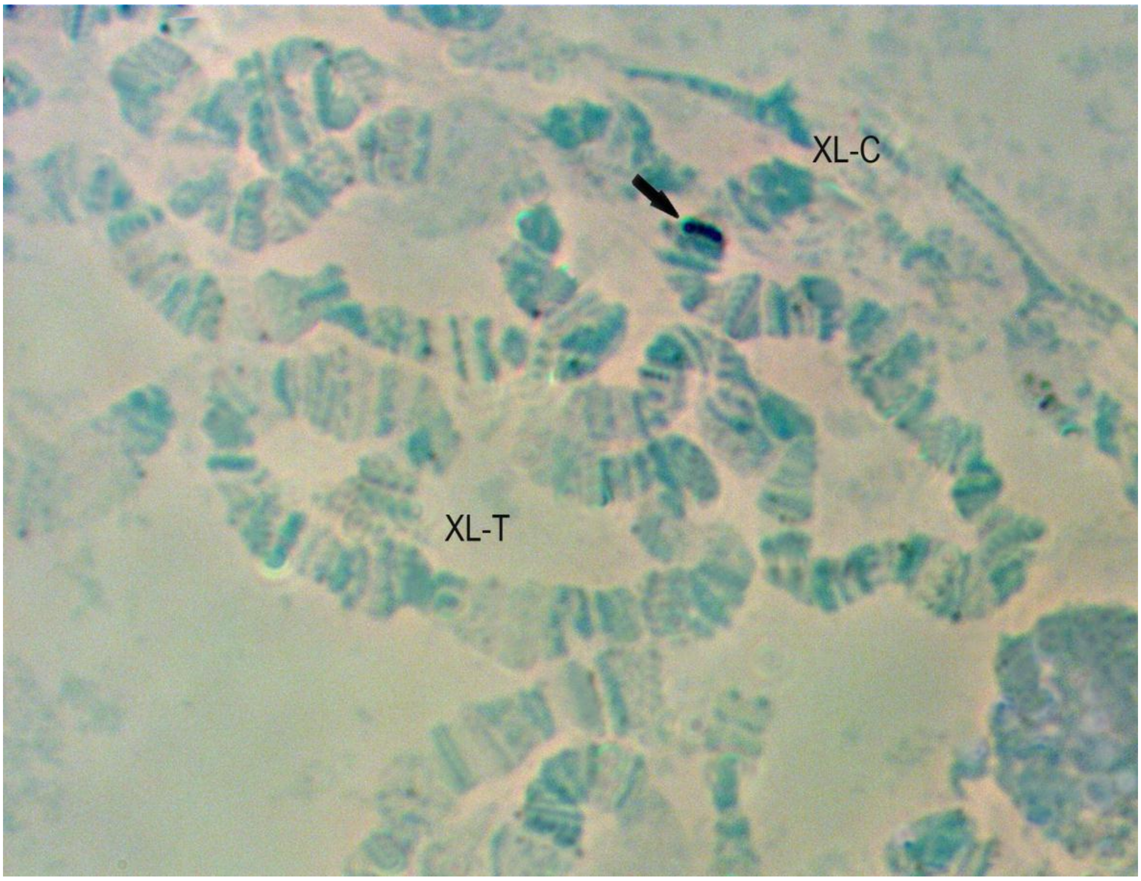

FIGURE S1 *In situ* hybridization of the *Dwil\GK16707* gene (scaffold 4963) to the *D. willistoni* chromosome XL arm**.** The black arrow indicates the hybridization signal site in section 1C. **XL-T:** XL arm telomere. **XL-C:** XL arm centromere.

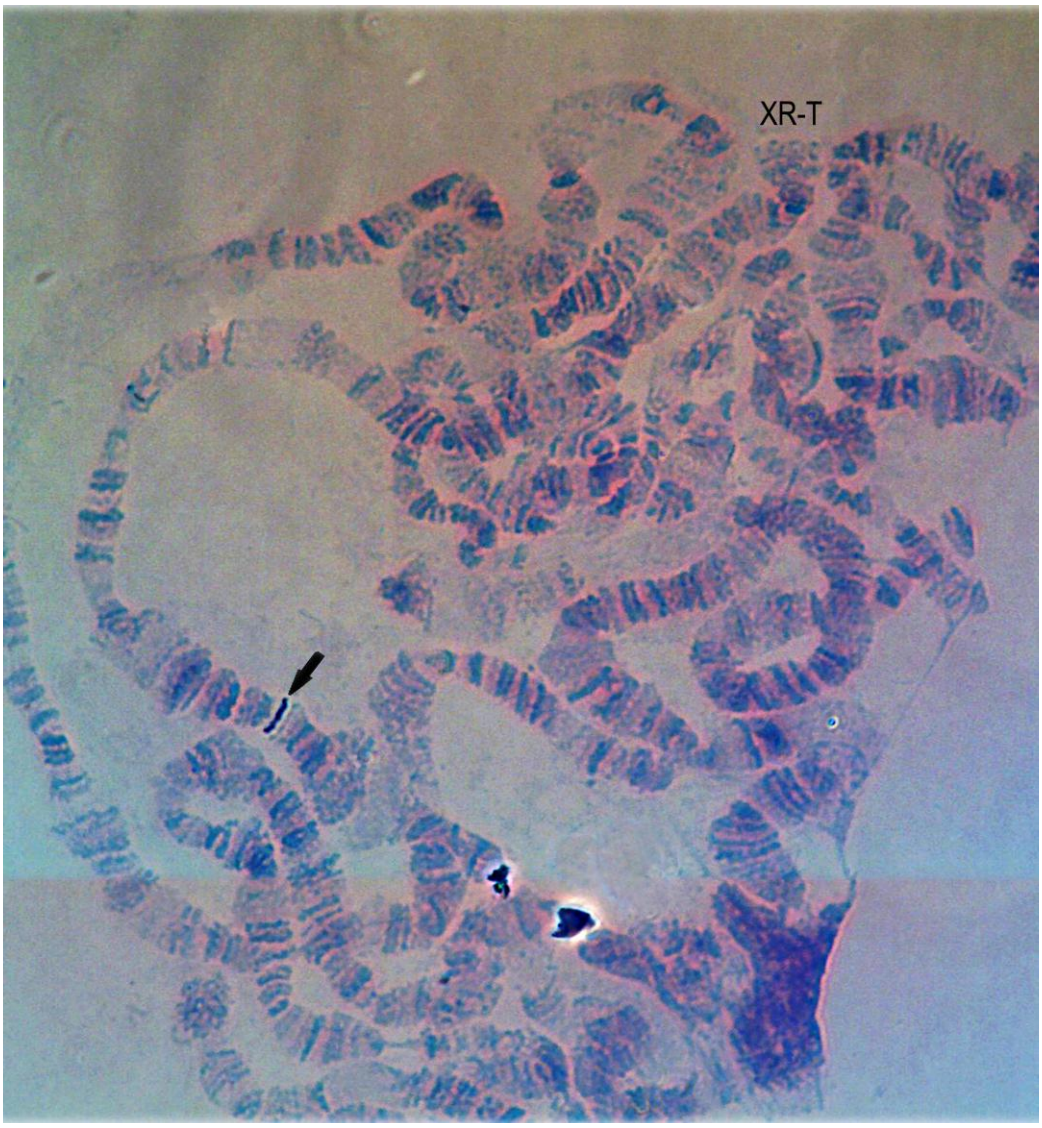

FIGURE S2 *In situ* hybridization of the *Dwil\GK17758* gene to the *D. willistoni* chromosome XR arm. This gene is located in the chimeric scaffold 4822, which was split into two Muller elements: a large portion in the IIR arm (see Table 1) and a smaller portion, containing the *Dwil\GK17758* gene, in the XR arm. The black arrow indicate hybridization signal site in section 27C. **XR-T:** XR arm telomere.

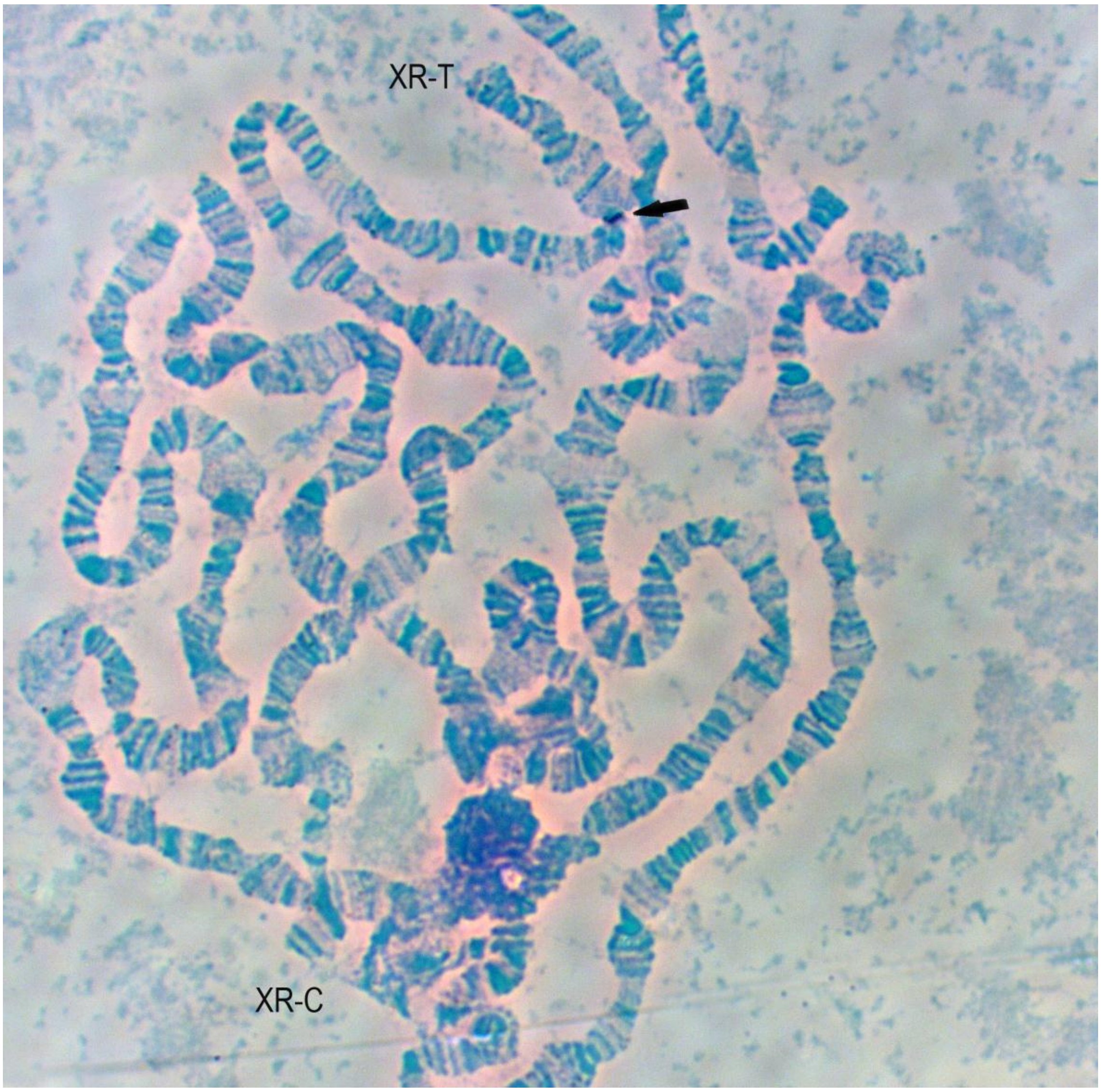

FIGURE S3 *In situ* hybridization of the *Dwil\GK16749* gene (scaffold 4511). This scaffold is the most telomeric in this chromosome arm. The black arrow indicates the hybridization signal in section 34B. **XR-T:** XR arm telomere. **XR-C:** XR arm centromere.

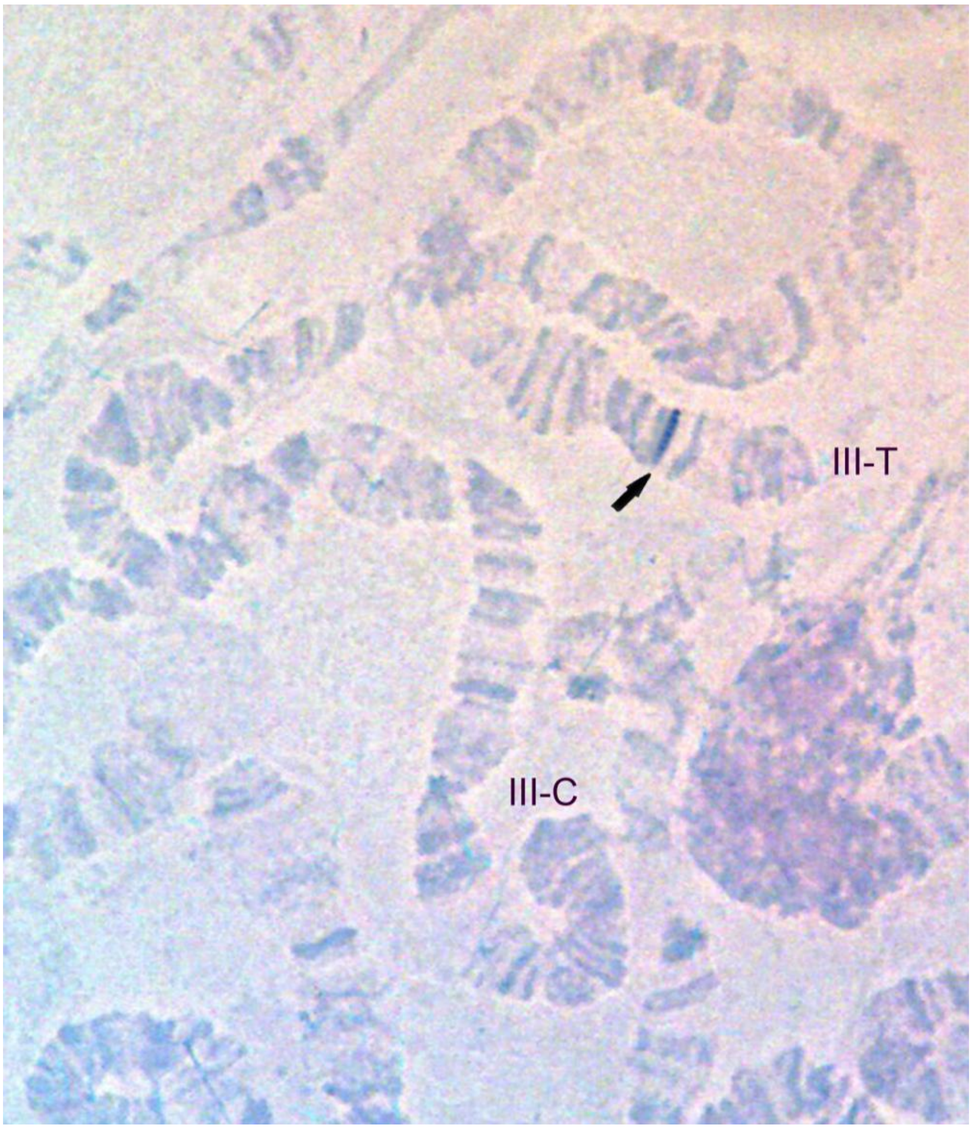

FIGURE S4 *In situ* hybridization of the *Dwil\GK22422* gene (scaffold 4921) to chromosome III. The black arrow indicates the hybridization signal in section 94D of this chromosome. This gene is located in the most telomeric scaffold (4921) and its cytological localization confirms its position in the scaffold. **III-T:** chromosome III telomere. **III-C:** chromosome III centromere.
