# Supplementary material for: Reassignment of Drosophila willistoni Genome Scaffolds to Chromosome II Arms
Source: G3 (Bethesda). 2015 Oct 4;5(12):2559–66. doi: 10.1534/g3.115.021311 (PMC4683629; doi:10.1534/g3.115.021311)
Supplement: Supporting Information [file supp_g3.115.021311_FigureS1.pdf]

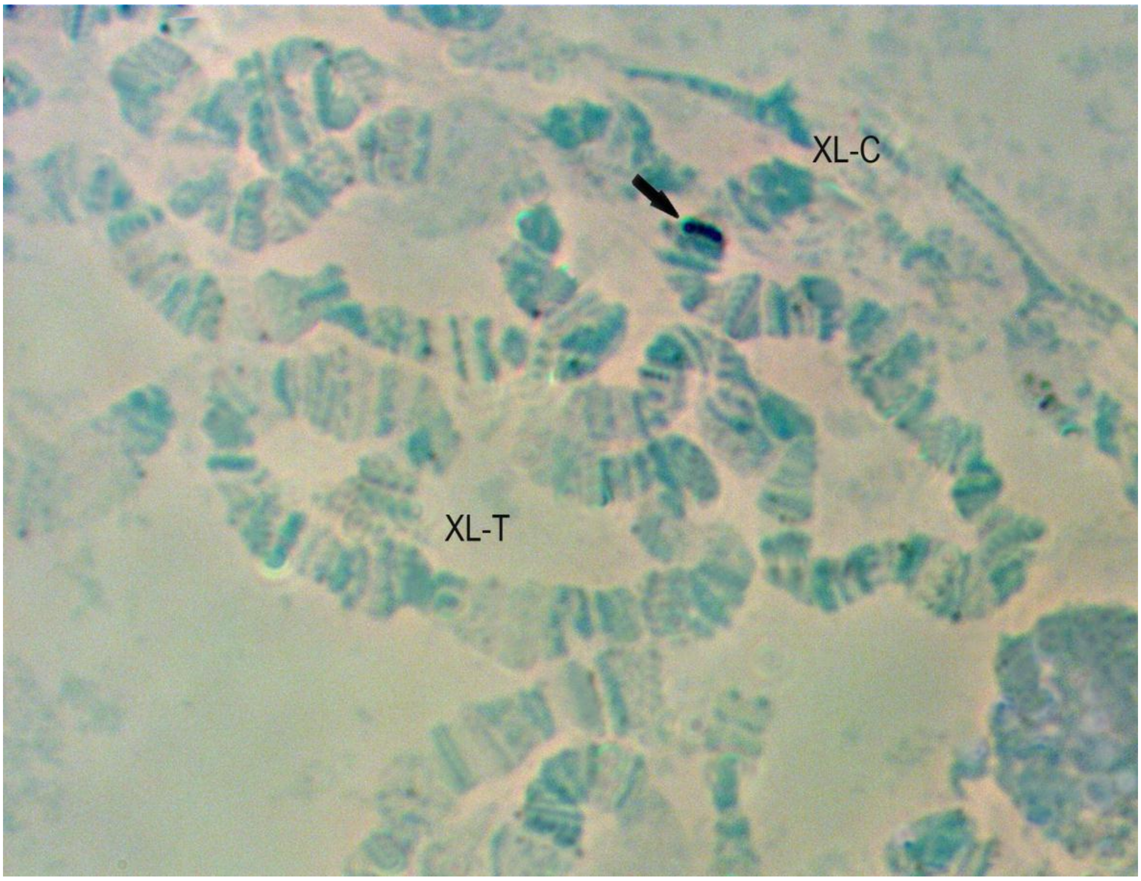

FIGURE S1 *In situ* hybridization of the *Dwil\GK16707* gene (scaffold 4963) to the *D. willistoni* chromosome XL arm**.** The black arrow indicates the hybridization signal site in section 1C. **XL-T:** XL arm telomere. **XL-C:** XL arm centromere.
