# Supplementary material for: Reassignment of Drosophila willistoni Genome Scaffolds to Chromosome II Arms
Source: G3 (Bethesda). 2015 Oct 4;5(12):2559–66. doi: 10.1534/g3.115.021311 (PMC4683629; doi:10.1534/g3.115.021311)
Supplement: Supporting Information [file supp_g3.115.021311_FigureS2.pdf]

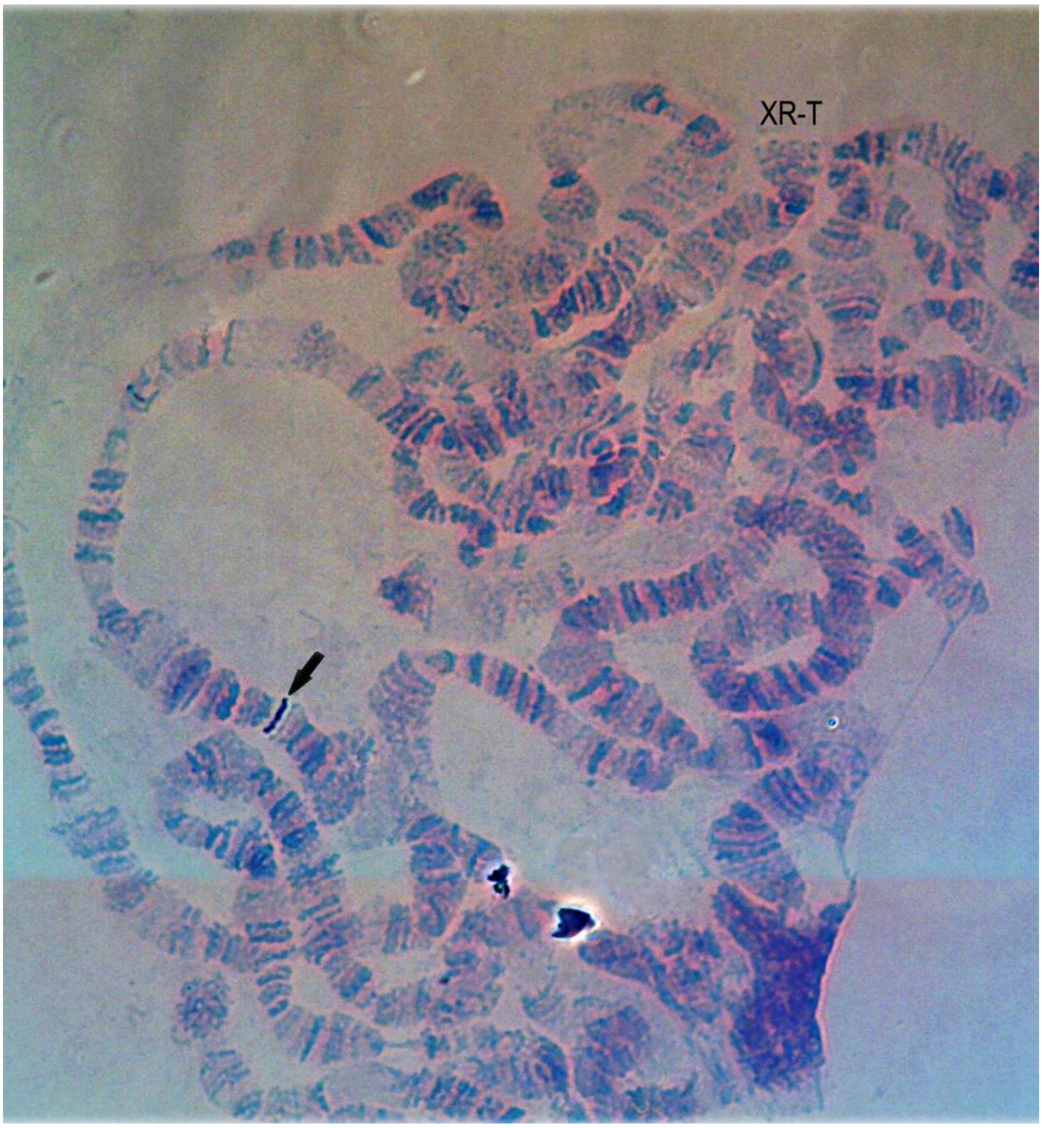

FIGURE S2 *In situ* hybridization of the *Dwil\GK17758* gene to the *D. willistoni* chromosome XR arm. This gene is located in the chimeric scaffold 4822, which was split into two Muller elements: a large portion in the IIR arm (see Table 1) and a smaller portion, containing the *Dwil\GK17758* gene, in the XR arm. The black arrow indicate hybridization signal site in section 27C. **XR-T:** XR arm telomere.
