# Supplementary material for: Reassignment of Drosophila willistoni Genome Scaffolds to Chromosome II Arms
Source: G3 (Bethesda). 2015 Oct 4;5(12):2559–66. doi: 10.1534/g3.115.021311 (PMC4683629; doi:10.1534/g3.115.021311)
Supplement: Supporting Information [file supp_g3.115.021311_FigureS3.pdf]

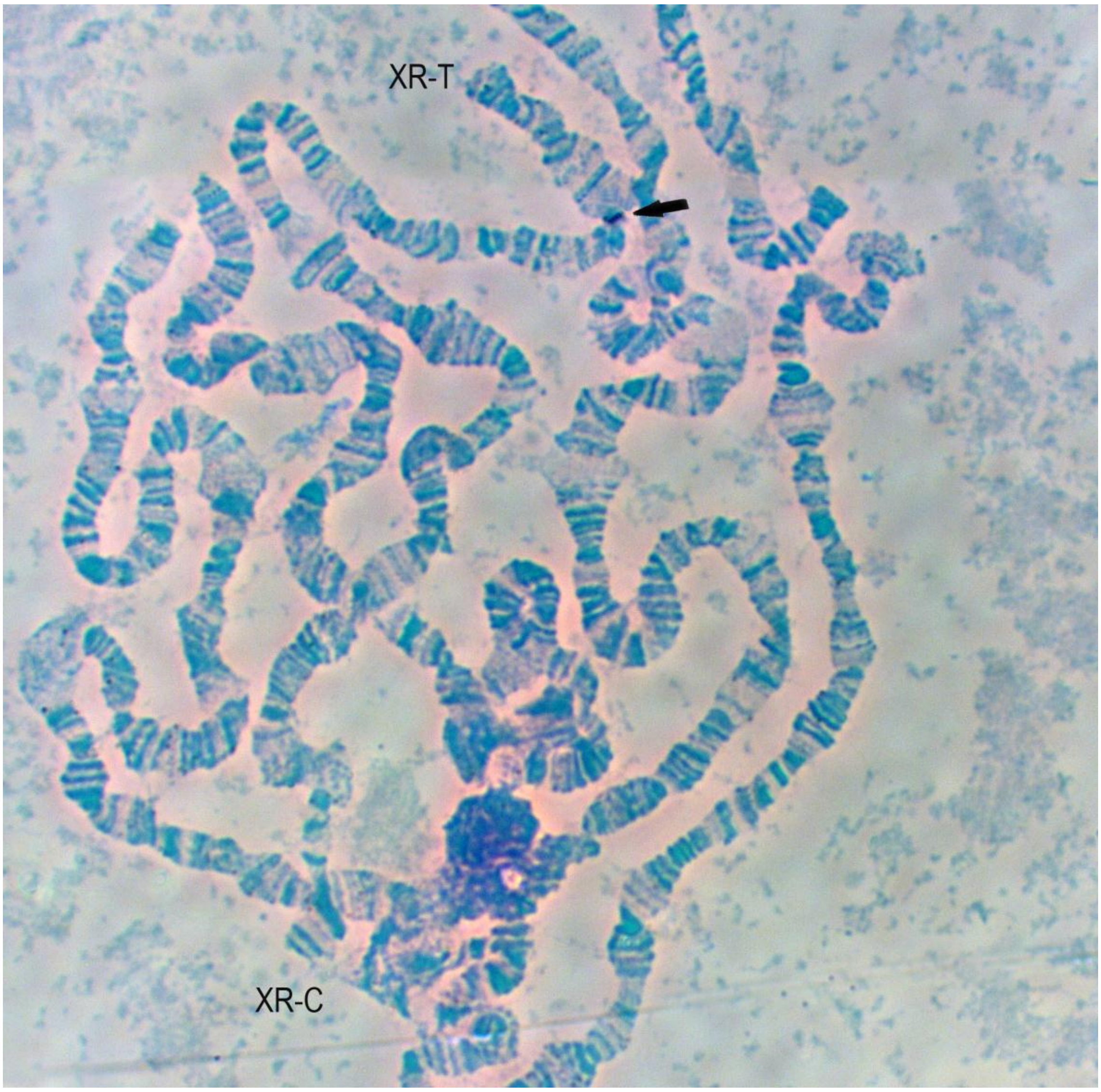

FIGURE S3 *In situ* hybridization of the *Dwil\GK16749* gene (scaffold 4511). This scaffold is the most telomeric in this chromosome arm. The black arrow indicates the hybridization signal in section 34B. **XR-T:** XR arm telomere. **XR-C:** XR arm centromere.
