# Supplementary material for: Reassignment of Drosophila willistoni Genome Scaffolds to Chromosome II Arms
Source: G3 (Bethesda). 2015 Oct 4;5(12):2559–66. doi: 10.1534/g3.115.021311 (PMC4683629; doi:10.1534/g3.115.021311)
Supplement: Supporting Information [file supp_g3.115.021311_FigureS4.pdf]

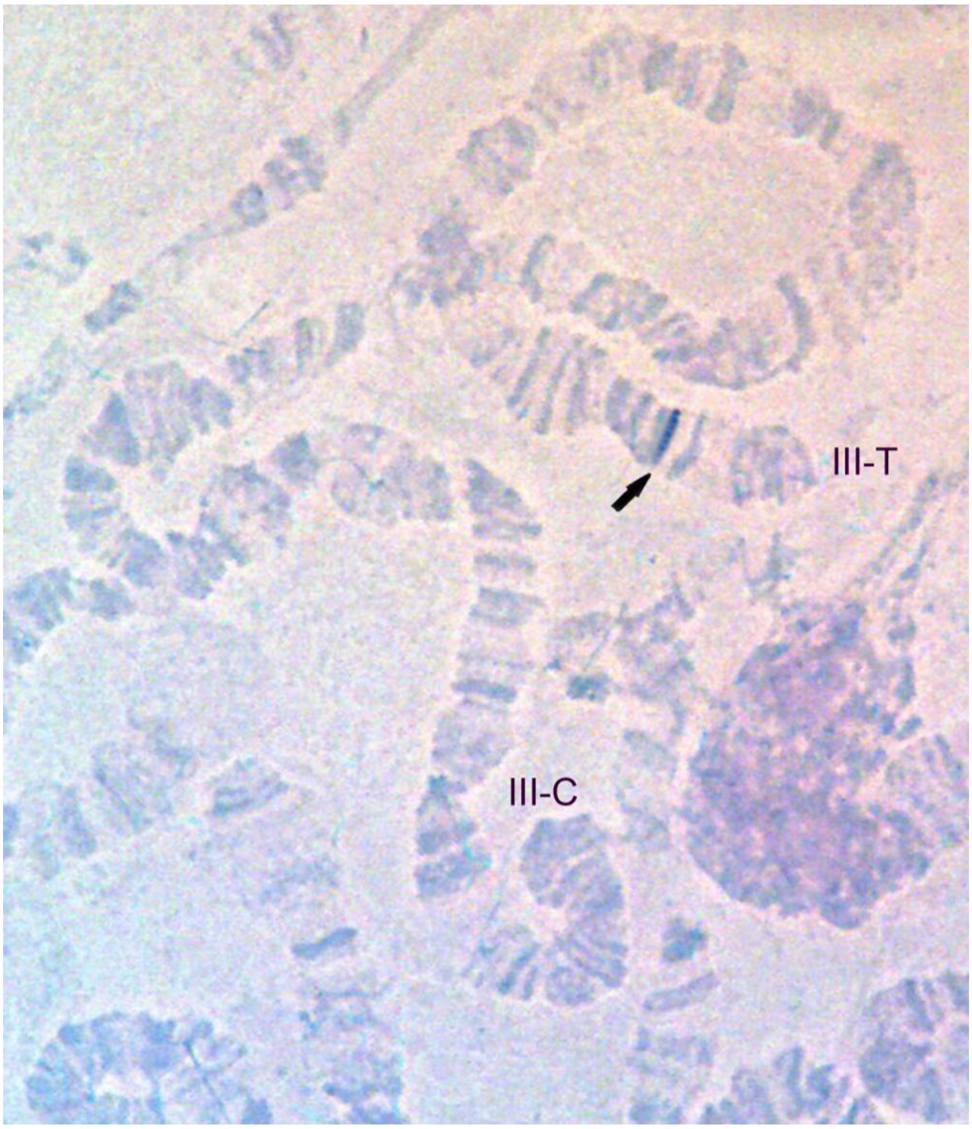

FIGURE S4 *In situ* hybridization of the *Dwil\GK22422* gene (scaffold 4921) to chromosome III. The black arrow indicates the hybridization signal in section 94D of this chromosome. This gene is located in the most telomeric scaffold (4921) and its cytological localization confirms its position in the scaffold. **III-T:** chromosome III telomere. **III-C:** chromosome III centromere.
