# Supplementary material for: Reassignment of Drosophila willistoni Genome Scaffolds to Chromosome II Arms
Source: G3 (Bethesda). 2015 Oct 4;5(12):2559–66. doi: 10.1534/g3.115.021311 (PMC4683629; doi:10.1534/g3.115.021311)
Supplement: Supporting Information [file supp_g3.115.021311_TableS1.pdf]

Table S1 Gene markers used for chromosomes X and III of *Drosophila willistoni*. The scaffold number corresponds to the last four numbers of the scaffolds, which all start with scf2_110000000. The scaffold number and scaffold position of genes corresponds to the material available in the FlyBase database (St. Pierre *et al*. 2014).

***D. willistoni* Gene**	***D. melanogaster* Ortholog Gene**	**Scaffold number**	**Scaffold position of gene**	**Cytological position/chromosome**	**Primers F and R (5'-3')**
Dwil\GK16707	*Dmel\unc*	4963	432,088..435,746	1C/XL arm	ACTCAGTCTTCGACGGAAGC<br>AGTTGTATCGGATTCTACCA
Dwil\GK17758	*Dmel\ida*	4822	3,033,141..3,041,719	27C/XR arm	GCTGCATTAGATCCTCATAG<br>GGCAGCCAACAGTCCATACA
Dwil\GK16749	*Dmel\CG13313*	4511	7,841,949..7,843,999	34B/XR arm	GCTATCAGTCACCGTGTAGA<br>GGCAGTTGCTCCACCATCAC
Dwil\GK22422	*Dmel\CG31204*	4921	3,260,674..3,262,239	99D/chromosome III	GAGTCAATGCGTCCATACCA<br>GGATAATCCTCACGAGACTG
